# Chats about New Books

**Published:** 1887-08-13

**Authors:** 


### CHATS ABOUT NEW BOOKS.

[Books for Review should be sent to the Editor, The Lodge, Porchester Square, W.]

### MARRYING & GIVING IN MARRIAGE.*

Mrs. Molesworth in this book tries to present us with some real personages, and succeeds well. Some of them are real enough to make us angry, and others to excite partly our sympathy and perhaps partly our contempt. Contempt, however, is a cheap sentiment, and indicates quite as often a want of patience and kindliness in the person who feels it as a want of worth in the object of it. The experienced man, therefore, who desires to be just and kindly towards his fellows is always on his guard against the indulgence of the feeling. The heroine, Aveline Verney, is one of the gentlest, simplest, and most lovable of women. She is made for no other purpose than to be the co-mate of a chivalrous and noble gentleman. Unhappily, such gentlemen are not very plentiful in any case, and they are still more difficult to find if money be demanded along with their other desirable qualities.

As a set-off to Aveline, the Lady Christina, her mother, is brought into relief. Poor Lady Christina! She is a woman with ten times the force of character possessed by her daughter, and not at all bad or disagreeable naturally. But Satan has tempted her through her butcher's and baker's bills, and she has fallen from her high estate as a dignified and pleasant gentlewoman into a condition of petty pride and sordid meanness.

The old story is retold. Aveline loves Mr. Hereward, who is well worthy to be loved, and her marriage with him would make her the happiest of women. But alas! alas! alas! He is like ten thousand other men of the highest possible degree of moral and intellectual merit—poor. Why are there no heroic means whereby a man of heroic mould may suddenly become rich? Why does the law object to his turning freebooter, and storming the castle of some wicked baron and reigning in his stead? International law does not object if the wicked baron happens to be a feeble king, and his necessitous neighbour a bigger and stronger king. These things are not well managed even in novels.

Now the villain of the piece, young Ayrton—who surely must be drawn from life, he is such a detestable specimen of an unlicked cub—has all he can possibly need in the way of money, and abundance more in prospect. Taking his character into consideration with those of his parents, we cannot help feeling that Mrs. Molesworth hardly occupies the modern scientific position on the subject of heredity. Perhaps she is old enough to know that many scientific and semi-scientific people adopt curious fads, and by-and-by lay them down again. She probably, therefore, likes to travel on the good old-fashioned road of every-day experience. Perhaps she is right. At any rate, although Ayrton's father is an honourable gentleman, and his mother a harmless lady, Ayrton himself is a man whom one would be ashamed even to kick. All through the book the reader is in a state of mortal terror lest Mrs. Molesworth should really allow this disreputable stable-man to marry poor Aveline. Aveline herself is so annoyingly wanting in backbone, so overwhelmingly anxious to please her mother; and the mother continues from beginning to end so hopelessly unable to see anything in the world but the miserable money, that the suspense is as agonising as the most exacting young lady-reader can wish it to be.

Is the book a true picture of life? There is more than a possibility that it is. If it be, one cannot help feeling both sad and disgusted. Money is, no doubt, a great power and a great convenience. It is more indispensable a thousand times than rank or birth, or any of the other things men so greedily long after. But if ever it becomes to the majority of thinking and capable Englishmen the highest of all objects of desire, then undoubtedly England will have begun her downward course. Mrs. Molesworth has done her best to show that "virtue and intelligence are better than gold, yea, than much fine gold"; and she has not laboured unsuccessfully.

* By Mrs. Molesworth. London: Longmans, Green & Co.

### PRESENTATION TO MR. P. MICHELLI.

After the weekly meeting of the Board at St. Mary's Hospital on Friday, 29th July, a handsome tea and coffee service, of sterling silver, the gift of upwards of sixty of those most actively associated with the government of the institution, was presented in the Board Room by Sir James Pycroft, K.C.S.I., on their behalf, to Mr. Pietro Michelli, as an expression of the regard in which he was held by all connected with the Charity, and as a mark of their high sense of the value of the services rendered by him to St. Mary's Hospital during the five years he was Secretary. Among those present were Sir Edward Sieveking, M.D. LL.D., Colonel F. G. Bird, Rev. E. S. Dewick, Dr. Felce, Dr. Randall, Dr. Phillips, Messrs. C. Bird, Geo. P. Field, Joseph Freeman, R. H. Jackson, Roche Lynch, Malcolm Morris, A. G. Packe, H. W. Page, Alfred Scorer, and E. Parker Young. In making the presentation Sir James Pycroft said he acted on behalf of more than sixty persons, including members of the hospital staff, the Medical School, the various committees of governors, non-official and various others, more or less intimately connected with the hospital. During Mr. Michelli's tenure of the office the hospital had been eminently prosperous. Every department was in good state and order, the finances were improved, and the hospital had steadily risen in public estimation. Mr. Michelli is to be sincerely congratulated on this marked and most gratifying evidence of the estimation in which he has been and still is held by his late colleagues and fellow-workers.

